# Match-play movement and metabolic power demands of elite youth, sub-elite and elite senior Australian footballers

**DOI:** 10.1371/journal.pone.0212047

**Published:** 2019-02-28

**Authors:** Stephen J. Kelly, Mark L. Watsford, Michael J. Rennie, Rob W. Spurrs, Damien Austin, Matthew J. Pine

**Affiliations:** 1 Sydney Swans Football Club, Sydney, Australia; 2 Faculty of Health, University of Technology Sydney, Sydney, Australia; 3 Brisbane Lions Football Club, Brisbane, Australia; Nottingham Trent University, UNITED KINGDOM

## Abstract

**Aims:**

Currently minimal research has quantified physical requirement differences in match-play between youth and senior Australian football players. The aim of the current research was to describe and compare the movement profiles and energy cost of youth, sub-elite and elite senior Australian football match-play.

**Methods:**

Fifty-seven Australian footballers playing in an elite senior 20, sub-elite senior 16 and elite youth competition 21 participated in this study. Distance, speed based indices and metabolic power measures recording via Global Positioning System (GPS) devices were compared across three competition tiers. Kicks and handballs were collected via a commercial statistics provider (Champion Data) and compared across the competition tiers.

**Results:**

Youth players recorded less field time (elite: ES = 1.37/sub-elite: ES = 1.68), total distance (elite: ES = 1.64 /sub-elite: ES = 1.55) and high speed running (elite: ES = 0.90/sub-elite: ES = 0.26) compared to the elite and sub-elite players. The average energy cost of elite (ES = 2.19) and sub-elite (ES = 1.58) match-play was significantly higher that youth match-play.

**Conclusions:**

A progressive increase regarding physical demands was evident across AF competition tiers. The findings suggest that sub-elite match-play can provide a viable pathway for youth players to develop physical capacity and technical skills before transitioning to elite senior match-play.

## Introduction

Team sports movement demand information is commonly collected by video based analysis and more recently global positioning systems (GPS) and accelerometer based microtechnology. This has provided quantitative data relating to training and match workloads within field sports. This data has recently been integrated with technical skills, providing holistic insights into player performance [1–3]. Physical performance within Australian football (AF) match-play has previously been reported for elite senior (Australian Football League) [4, 5], sub-elite senior (state reserve competition league) [6, 7] and elite youth players (national under 18 year competition) [3, 8, 9]. Despite this body research with AF, there are minimal objective studies has directly compared across competition tiers from youth through to elite senior players [8]. With the evolving nature of professional AF match-play [6, 8], challenges persist regarding optimal development and management strategies for youth players. Therefore, contemporary data is required to guide best practices regarding the youth development pathway within AF.

Previous research investigating the gap in match-play demands between youth and professional AF players reported a divergence in match-play intensity between these competition tiers over a number of competitive seasons [8]. Elite senior AF match-play is more intense and comprises of greater high speed running and higher energy expenditure than sub-elite senior match-play [6, 7, 10]. From a technical skills perspective, it appears that senior players classified as lower calibre perform at a high intensity during match-play [2]. This poses a number of challenges regarding the optimisation of physical and technical skill development of youth and sub-elite players. Although a 1 to 4 year period is typically required before players make their debut within AF, there is increasing evidence that newly drafted players are exposed to professional match-play earlier in their careers [8]. This is likely accelerated when players are recruited higher during the national draft selection. There may be risks that young players have not acquired adequate physical readiness to cope with the increased physical demands of professional AF [8]. Inferior strength and movement competency is evident between youth AF players [11–13], highlighting potential injury incidences for younger players exposed to an increased training workload or match-play intensity. Therefore, the benefits of quantifying differences in physical demands between youth, sub-elite senior and elite senior AF match-play are twofold. Firstly, it can direct training practices within the youth development pathway and secondly provide a framework to optimally prepare youth players for professional AF match demands.

While a broad understanding of the physiological and anthropometric characteristics of youth AF players is known [14–16], evidence-based research is required to directly quantify differences across competition tiers in AF. Therefore, this study aimed to describe and compare match-play movement demands, energy cost and the number of kicks and handballs involvements across three AF competition tiers. Given the differences between the physical demands of elite and sub-elite match-play have been widely examined [6, 7, 17], this research was primarily undertaken to identify differences between [1] youth and elite senior players and [2] youth and sub-elite senior players. A progressive increase in match-play intensity across three AF competition tiers was hypothesised. Specifically, more high speed running (HSR), high accelerations (HA), high decelerations (HD) and a higher match-play energy cost would be evident in the elite and sub-elite senior competition tiers. Given professional AF players will likely have greater technical skill proficiency, it was expected that elite and sub-elite senior players would accrue more kick and handball involvements.

## Materials and methods

### Study design

To investigate differences in match-play movement demands and metabolic power indices across three AF competition tiers, this cross-sectional study was undertaken during one competitive in-season period. The study involved 17 elite senior, 17 sub-elite senior and 8 national U18 competition games. Data files were delimited to players classified as nomadic and other rotating positions, to standardize the collection procedures [4]. Team selection varied weekly according to injury status and player form, with data analysis undertaken on players who participated in >75% of the total field time [4]. Exclusion criteria included fixed forward, back and ruck positions, injuries to players and data files that were <74.99% of the total field time.

### Participants

A total of 57 players from three tiers of AF participated within this research study. The distribution of players included; 20 elite senior (24.9 ± 3.8 years, 87.8 ± 9.4 kg, 185.2 ± 6.9 cm), 16 sub-elite senior (21.7 ± 2.7 years, 84.9 ± 5.5 kg, 184.6 ± 6.1 cm) and 21 youth players (17.9 ± 1.6 years, 80.8 ± 4.1 kg, 182.7 ± 4.2 cm). Written informed consent was obtained from participants and the organisations involved, with ethical approval granted by the Human Research Ethics Committee at the University of Technology Sydney (UTSHREC: 2014000427).

### Data analysis

Movement demands were recorded using commercially available 10Hz GPS devices (Catapult Innovations, Melbourne Australia), with players assigned the same device for each match, thus ensuring heightened reliability within the data [4]. The reliability and validity of these GPS devices have been reported elsewhere and deemed acceptable for data pooling [18, 19]. The distribution of 467 data files included; 237 elite senior, 103 sub-elite senior and 77 youth competition files. Devices were placed in a custom designed pouch, positioned between the scapulae within the player's jersey as previously described [4]. Players were familiarised with these protocols, with individual devices assigned over the data collection period to minimize between-unit variability [20].

Following each match, data was downloaded using Catapult Sprint (version 5.1.6; Catapult Innovations), with bench time and time between quarters excluded via the manufacturer’s software. All data was exported to Microsoft Excel (Microsoft 2010, Redmond, USA) for further analyses. Non-individualised speed, acceleration and deceleration zones were established from classifications used in previous research [7, 21]. Variables were expressed per minute of game time due to player rotations, providing individual total field times.

Movement demand and workload variables documented in previous AF studies were examined [4, 7], including total distance, average speed per minute and Playerload^TM^ per minute. Velocity based measures were examined for distance, time spent and the number of efforts in two zone classifications, including low speed running (LSR, 0.0–14.4 km^.^h^-1^) and high speed running (HSR, 14.4–20.0 km^.^h^-1^) [7]. Distance, time spent and number of efforts within acceleration and deceleration zone classifications were investigated according to previous research [21] for low decelerations (LD, -0.65 to -1.46m^.^s^-2^), moderate decelerations (MD, -1.47 to -2.77m^.^s^-2^), high decelerations (HD, <-2.78 m^.^s^-2^), low accelerations (LA, 0.65 to 1.46m^.^s^-2^), moderate accelerations (MA, 1.47 to 2.77m^.^s^-2^) and high accelerations (HA, >2.78 m^.^s^-2^).

Metabolic power variables analysed from the 10Hz GPS devices, ensured only data containing the highest resolution [22]. These variables were calculated using an algorithm established previously [23] and included average energy expenditure (kJ^.^kg^-1^), estimated distance and total time and number of efforts in low power (LP, 0–19.99 W^.^kg^-1.^min^-1^), high power (HP, 20–39.99 W^.^kg^-1.^min^-1^) and very high power (HP, >40 W^.^kg^-1.^min^-1^) zone classifications [7]. Total kicks (number of times a player disposed of the ball by foot) and handballs (number of times a player disposed of the ball by hand) involvements were obtained from the provider of match statistics for the Australian football league (AFL) (Champion Data, Victoria, Australia) and analysed per minute of game time.

### Statistical analysis

Descriptive statistics for all variables were reported as mean and 95% CI (lower and upper), with data assessed for normality and sphericity. A linear mixed model was used to account for pseudo-replication within the dataset. Individual players were included as a random effect, while level of competition was defined as a fixed effect. Bonferroni post hoc comparisons were used to identify differences between groups. The analysis was undertaken using Statistical Package for Social Sciences (version 21, IBM, Armonk, NY). Standardised effect size (ES) were computed and interpreted as <0.2, trivial; 0.21–0.6, small; 0.61–1.20, moderate; 1.21–2.0 large; >2.1 very large [24]. An alpha level of *P*<0.05 was used for all statistical tests.

## Results

Results for velocity, workload and technical skill variables across the competition tiers are reported in Table 1. Both senior player groups played more field time (ES = 1.37/1.68) and covered a great total distance (ES = 1.55/1.63) during match-play. Interestingly, when total distance (ES = 0.11/0.15) and Playerload^TM^ (ES = 0.21/0.53) were analysed relative to game time, differences between the youth players and senior player groups diminished. The senior player groups also performed more HSR distance (ES = 0.26/0.90), time (ES = 0.35/1.01) and efforts (ES = 0.32/1.06). Regarding technical skill measures, notable differences were solely evident for handballs (ES = 0.40) between sub-elite senior and youth players.

**Table 1:** Physical performance and technical skills for elite junior, elite and sub-elite Australian footballers. Values are mean (95% CI).

	Elite junior	Elite	Elite Junior vs Elite (Effect Size d)	Sub-Elite	Elite junior vs Sub-Elite (Effect Size d)
**Field Time**	85.66 (83.55–87.77)	101.12 (100.08–102.16)^a^	1.37	106.35 (104.84–107.87)^a^^b^	1.68
**Total Distance Covered (m)**	10940.22 (10661.11–11219.53)	13193.14 (13047.40–13339.81)^a^	1.64	13189.34 (12967.64–13412.72)^a^	1.55
**Average Speed (m.min**^**-1**^**)**	125.72 (122.73–128.70)	130.68 (129.28–132.08)^a^	0.15	126.53 (124.37–128.69)^b^	0.11
**Player Load**·**min**^**-1**^	12.39 (11.95–12.82)	13.74 (13.53–13.94)^a^	0.53	13.03 (12.71–13.35)^a^^b^	0.21
**Low Speed Distance Covered (m**·**min**^**-1**^**)**	89.04 (86.31–91.76)	86.41 (85.16–87.67)	0.62	89.77 (87.80–91.75)^b^	0.38
**High Speed Distance Covered (m**·**min**^**-1**^**)**	24.38 (22.67–26.10)	30.47 (29.62–31.32)^a^	0.90	25.64 (24.30–26.97)^b^	0.26
**Low Speed Time (%**·**min**^**-1**^**)**	89.09 (88.35–89.83)	85.48 (85.11–85.86)^a^	0.96	87.92 (87.34–88.50)^a^^b^	0.30
**High Speed Time (%**·**min**^**-1**^**)**	8.52 (7.93–9.11)	10.95 (10.65–11.24)^a^	1.01	9.13 (8.66–9.59)^b^	0.35
**High Speed Efforts (n**·**min**^**-1**^**)**	1.50 (1.42–1.57)	1.79 (1.75–1.83)^a^	1.06	1.51 (1.46–1.57)^b^	0.32
**Kicks (n**·**min**^**-1**^**)**	0.10 (0.09–0.11)	0.11 (0.10–0.11)	0.14	0.11 (0.10–0.11)	0.43
**Handballs (n**·**min**^**-1**^**)**	0.07 (0.06–0.08)	0.09 (0.08–0.09)	0.20	0.10 (0.09–0.10)^a^	0.40

^a^ Significantly different to elite junior AFL players (*P*<0.05).

^b^ Significantly different to elite AFL players (*P*<0.05).

Data pertaining to accelerations, decelerations and metabolic power indices are presented in Tables 2 and 3. The elite senior players performed more distance (ES = 0.67/1.98) and spent more time (ES = 0.16/1.68) undertaking accelerations and decelerations. Conversely, youth players recorded a greater number of moderate and high acceleration (ES = 0.35/1.00) and deceleration (ES = 0.60/0.63) efforts. It was clear that mean energy cost of elite (ES = 2.19) and sub-elite match-play (ES = 1.58) was considerably higher than youth match-play. Additionally, the estimated distance covered during elite (ES = 1.73) and sub-elite (ES = 1.12) match-play was considerably higher than youth match play. Finally, the youth players spent less time and recorded fewer efforts within low to high metabolic power zones, with differences ranging from small to very large.

**Table 2:** Acceleration and deceleration data for elite junior, elite and sub-elite Australian footballers. Values are mean (95% CI).

	Youth	Elite Senior	Elite Junior vs Elite (Effect Size d)	Sub-Elite Senior	Elite junior vs Sub-Elite (Effect Size d)
**Distance Covered (m**·**min**^**-2**^**)**					
High Decelerations	1.16 (1.10–1.22)	1.34 (1.31–1.37)^a^	0.71	1.14 (1.10–1.18)^b^	0.05
Moderate Decelerations	3.39 (3.27–3.51)	3.69 (3.63–3.75)^a^	0.80	3.33 (3.24–3.42)^b^	0.32
Low Decelerations	7.66 (7.33–7.98)	10.43 (10.26–10.61)^a^	1.96	8.44 (8.19–8.69)^a^^b^	0.90
Low Accelerations	8.80 (8.45–9.16)	11.72 (11.54–11.90)^a^	1.98	9.86 (9.62–10.11)^a^^b^	0.99
Moderate Accelerations	4.12 (3.97–4.27)	4.68 (4.60–4.75)^a^	0.98	4.18 (4.07–4.28)^b^	0.42
High Accelerations	1.66 (1.56–1.75)	1.92 (1.87–1.96)^a^	0.67	1.38 (1.31–1.45)^a^^b^	0.43
**Time Spent (%**·**min**^**-2**^**)**					
High Decelerations	0.86 (0.80–0.93)	1.25 (1.21–1.28)^a^	1.32	0.86 (0.81–0.92)^b^	0.17
Moderate Decelerations	2.34 (2.27–2.41)	2.44 (2.40–2.47)^a^	0.44	2.28 (2.22–2.33)^b^	0.19
Low Decelerations	5.71 (5.56–5.87)	6.79 (6.70–6.88)^a^	1.68	6.02 (5.90–6.13)^a^^b^	0.89
Low Accelerations	5.65 (5.49–5.82)	6.70 (6.61–6.80)^a^	1.60	6.12 (5.99–6.23)^a^^b^	0.59
Moderate Accelerations	2.56 (2.48–2.64)	2.63 (2.58–2.67)	0.16	2.60 (2.53–2.66)	0.54
High Accelerations	1.27 (1.20–1.35)	1.46 (1.42–1.50)^a^	0.46	1.12 (1.07–1.18)^a^^b^	0.14
**Number of Efforts (n**·**min**^**-2**^)					
High Decelerations	0.55 (0.52–0.58)	0.46 (0.44–0.47)^a^	0.63	0.52 (0.49–0.54)^b^	0.38
Moderate Decelerations	1.01 (0.94–1.06)	0.86 (0.83–1.01)^a^	0.60	0.98 (0.93–1.02)^b^	0.25
Low Decelerations	2.82 (2.71–2.93)	3.26 (3.19–3.33)^a^^b^	1.30	2.95 (2.87–3.02)	0.80
Low Accelerations	2.81 (2.69–2.93)	3.24 (3.16–3.31)^a^	1.12	3.09 (3.02–3.17)^a^	0.85
Moderate Accelerations	1.04 (1.01–1.12)	0.95 (0.91–0.99)^a^	0.35	1.12 (1.07–1.17)^a^	0.50
High Accelerations	0.49 (0.46–0.52)	0.36 (0.35–0.37)^a^	1.00	0.38 (0.36–0.40)^a^	0.58

^a^ Significantly different to elite junior AFL players (*P*<0.05).

^b^ Significantly different to elite AFL players (*P*<0.05).

**Table 3:** Metabolic power data and energy cost for elite junior, elite and sub-elite Australian footballers. Values are mean (95% CI).

	Youth	Elite Senior	Youth vs Elite Senior (Effect Size d)	Sub-Elite Senior	Youth vs Sub-Elite (Effect Size d)
**Peak Metabolic Power (W**·**kg**^**-1**^**)**	195.48 (189.63–210.32)	168.74 (162.36–175.23)^a^	0.41	165.29(155.48–175.10)^a^	0.19
**Average Energy Expenditure (kJ**·**kg**^**-1**^**)**	47.81(46.38–49.22)	62.55 (61.85–63.15^a^	2.19	59.61 (58.57–60.65)^a^^b^	1.58
**Estimated Distance (ED)**	13856 (13469–14244)	16959 (16788–17130)^a^	1.73	16373 (16099–16646)^a^^b^	1.12
**Estimated Distance Index**	1.269 (1.262–1.276)	1.283 (1.280–1.287)^a^	0.49	1.248 (1.242–1.253)^a^^b^	0.27
**Time Spent (W**·**kg**^**-1.**^**min**^**-1**^**)**					
Low Power	73.06 (71.28–74.84)	82.73 (81.85–83.60)^a^	0.91	90.91 (89.55–92.28)^a^^b^	1.45
High Power	7.10 (6.67–7.53)	10.31 (10.08–10.54)^a^	1.76	8.87 (8.52–9.23)^a^^b^	1.04
Very High power	1.85 (1.70–1.99)	2.71 (2.63–2.77)^a^	1.57	2.15 (2.04–2.26)^a^^b^	0.67
**% Time Spent (W**·**kg**^**-1.**^**%min**^**-1**^**)**					
Low Power	85.51 (84.81–86.22)	82.25 (81.88–82.63)^a^	1.16	85.86 (85.27–86.44)^b^	0.23
High Power	8.00 (7.51–8.49)	10.29 (9.99–10.47)^a^	1.18	8.58 (8.20–8.95)^b^	0.38
Very High power	2.05 (1.91–2.19)	2.67 (2.60–2.74)^a^	1.10	2.06 (1.95–2.17)^b^	0.10
**Number of Efforts (W**·**kg**^**-1.**^**n**·**min**^**-1**^**)**					
Low Power	353.75 (340.49–367.01)	508.83 (501.57–516.08)^a^	2.37	475.78 (464.66–486.91)^a^^b^	1.60
High Power	158.46 (150.75–166.17)	223.36 (219.38–227.34)^a^	1.96	195.99 (189.81–202.16)^a^^b^	1.18
Very High power	56.34 (51.56–61.13)	81.47 (79.07–83.86)^a^	1.38	66.08 (62.31–69.84)^a^^b^	0.64

^a^ Significantly different to elite junior AFL players (*P*<0.05).

^b^ Significantly different to elite AFL players (*P*<0.05).

## Discussion

This study provided a comparative analysis of match-play across three AF competition tiers. Match-play movement demand differences between elite senior and sub-elite senior competitions have been documented [6, 7], however, minimal research has incorporated youth AF players within the analysis [8]. The main findings of the present study revealed a higher total game time for elite senior and sub-elite senior match-play and an overall progressive increase in HSR, HA, HD and energy cost across the competition tiers. These findings provided similar outcomes to previously observed match-play data between competition tiers within AF [6, 7]. While physical performance differences were in congruence with the hypothesis, it appears that more kick and handball involvements are accrued in both sub-elite and elite match-play Therefore, these findings illustrate a potential match-play pathway for youth players transitioning to professional AF, through sub-elite competitions.

Overall elite senior match play had the greatest workload and involved more HSR than the other competition tiers. Similar findings were reported between youth and elite senior AF players over a number of competitive seasons. Youth game speed within this research remained similar over a five year period, while elite senior match-play yielded a 25% increase [8]. Elite senior players in the current investigation recorded 22%, 25% and 18% more HSR distance, time and efforts per minute, compared to the youth players. Alternatively, sub-elite players performed 5%, 7% and 1% more HSR distance, time and efforts per minute compared to the youth players. Importantly the capacity to perform HSR has been positively correlated with team selection in AF [25] and playing at a higher competition tier in futsal [26]. Conversely, previous AF research reported that higher caliber players as rated by skill coaches, performed less HSR than the lower caliber players within the same team. It was suggested that as a result of the increased field time, higher caliber players displayed increased fatigue [2]. Due to the greater running volumes and intensities in both senior competition tiers, it seems prudent that youth players concurrently develop the aerobic and anaerobic energy systems. Further investigation regarding training practices to improve these physical characteristics is therefore warranted.

The findings regarding kicks and handballs indicated that both sub-elite and elite senior match-play afforded an opportunity to develop these technical skills. The number of total team kicks compared to the opposition has been reported to be a key indicator of performance success with AF [27], highlighting the importance of developing this skill. Regarding the competition tiers, it is likely that the interplay between internal (experience, technical ability, decision making) and external (opposition, team tactics, game state) factors [1], influenced technical skill proficiency differently for each group. Investigations within AF [1] and soccer [28] match-play reported a strong relationship between the early onset of fatigue and a reduction in technical skill performance. Furthermore, alternative AF research reported higher caliber players accrued more skill involvements, while also undertaking less high intensity running. [2]. These players displayed a higher chronological age [2], indicating a relationship between technical skill acumen and match-play experience within AF. The current research suggests that youth players have not developed the required physical capacity to maintain skill proficiency during match-play.

While research has decribed the acceleration and deceleration profiles of sub-elite [6, 7] and elite AF [1, 10, 29, 30] players, minimal data is avaiable on youth AF players. Consequently little is known about the acceleration and deceleration profiles during elite youth AF match-play. Elite senior players in the current investigation covered 15% more distance and spent 13% to 36% more time performing HA and HD movements, compared to the youth players. Similar findings have been reported between elite and sub-elite senior AF players for distance and time spent in HA and HD zones [7]. This may reflect the superior capacity of elite senior players to perform and sustain moderate to high-intensity acceleration and deceleration movements. Interestingly, the youth players recorded 17% to 30% more HD and HA efforts compared to sub-elite and elite senior players. It seems youth players have developed a capacity to undertake HA efforts, however a diminished ability to sustain these rapid velocity changes. Due to the considerable energy cost of acceleration and deceleration movements [31], it is recommended that training practices with youth players replicate the various velocity, acceleration and deceleration movements evident in this research. Small-sided games are one such training modality utilised within soccer to stimulate match-play specific physical and technical skill development [32, 33]. The recent advent of metabolic power data derived from microtechnology has revealed that small-sided games elicit a high volume of acceleration and deceleration movements [33]. Small-sided games may replicate intense periods of match-play and therefore provide a beneficial training stimulus for AF players. Despite the regular implementation of this training modality within AF, little is currently known about the physical benefits of small sided games. Future research should measure how this training modality can positively develop the physical capacity of youth AF players.

Although the authors acknowledge the current conjecture regarding the validity of microtechnology to accurately quantify metabolic power [23, 34], this was one of the first studies to provide data on the energy cost of youth AF match-play. Both senior match-play tiers resulted in a substantially higher mean energy cost. Although the authors recognise these outcomes were likely biased by the greater field times evident for the senior competition tiers, these findings provide some practical implications for youth player development. The mean match-play energy cost for sub-elite and elite senior players was similar to a AF (57–66 kj^.^kg^-1^) [10] and soccer (66 kj^.^kg^-1^) [23] investigation. Conversely, a recent AF study reported a higher mean match-play energy cost ranging from 74–76 kj^.^kg^-1^ [7]. With the increased physical demands evident in elite senior AF [8], training practices within the youth develop pathway should continually evolve to prepare young players for the higher energy cost of senior match-play. As evident by the hierarchical trend in the current investigation, challenges clearly exist transitioning youth players to elite senior AF.

A notable strength of this investigation was the recruitment of one professional AF team (elite and sub-elite cohort) who were exposed to a homogeneous training environment, while also using an established and embedded elite youth development program. This provided the authors with a large cohort for a cross-sectional analysis. As with all match analyses, the findings within this investigation reflect the tactical decisions of the coaching staff during match-play, and therefore caution is warranted when extrapolating the results to alternative AF teams or competition tiers. This was one of the first studies to identify and compare movement demands from youth through to elite senior match-play, with many relevant outcomes established. Future research should examine specific training practices to prepare youth players transitioning to professional AF teams.

## Conclusion

A number of differences were evident for field time, HSR, HA, HD and match-play energy cost across competition tiers within AF. It was evident that sub-elite senior match-play allowed for increased field time, a reduction in running demands and similar technical skill involvements to elite senior match-play. Therefore, sub-elite senior match-play justifiably offers a match-play conduit to develop physical capacity and technical skill acumen during the transition from youth to elite senior AF match-play.
